# Ultrasound cracks the seed coat barrier: a strategy for whole-bean tempeh fermentation from black kidney bean with balanced protein-starch digestibility

**DOI:** 10.1016/j.ultsonch.2026.108113

**Published:** 2026-10-13

**Authors:** Yaohua Qin, Xinyu Qiang, Kun Wang, Jie He, Haijun Wang, Wei Li, Xin Rui

**Affiliations:** a College of Food Science and Technology, Nanjing Agricultural University, Jiangsu Province 211800, PR China; b College of Food Science, Heilongjiang Bayi Agricultural University, Heilongjiang Province 163319, PR China; c Agro-products Quality Safety and Testing Technology Research Institute, Guangxi Academy of Agricultural Sciences, Guangxi Province 530007, PR China

**Keywords:** Ultrasound treatment, Black kidney beans, Seed coat permeability, Tempeh, Protein bioaccessibility, Starch digestibility

## Abstract

Whole-bean tempeh production is hindered by the compact seed coat of black kidney beans, which restricts fungal colonization and nutrient bioaccessibility. Dehulling, the conventional solution, removes the nutrient-dense outer layer. This study employed ultrasound pretreatment (10–30 min) to modify seed coat structure prior to fermentation with *Rhizopus oligosporus*, aiming to overcome this barrier while preserving the seed coat. Ultrasound increased seed coat porosity from 0.9% to 4.5%, reduced hardness from 365.7 N to 73.5–116.8 N, and enhanced hydration, thereby facilitating fungal growth and enzyme activity. Ergosterol content, a marker of fungal biomass, increased 9.0-fold over the untreated control. SEC and CLSM further confirmed enhanced protein and starch degradation as well as cell wall disassembly in ultrasound-treated samples. Compared with untreated whole-bean tempeh, ultrasound-pretreated samples achieved 1.4–2.5-fold higher peptide levels, while maintaining moderate starch utilization distinct from the excessive degradation observed in dehulled tempeh. Protein bioaccessibility reached approximately 55%, bridging 37% of the digestibility gap between whole-bean and dehulled samples, yet the estimated glycemic index remained substantially lower (47.3–49.7) than that of dehulled tempeh (67.2). The 20-min treatment provided the best overall performance, demonstrating that controlled ultrasound is a viable strategy for producing nutritionally balanced whole-bean tempeh.

## Highlights

• Ultrasound disrupts black kidney bean seed coats, avoiding dehulling nutrient loss.
• Seed coat porosity rises from 0.9% to 4.5%, boosting fungal growth and fermentation.
• Ultrasound enhances protein bioaccessibility to ∼55%, closing a 37% gap to dehulled.
• 20-min ultrasound optimally balances fermentation efficiency and seed coat retention.

## 1 Introduction

Tempeh is a traditional fermented food produced by *Rhizopus* spp., valued for its characteristic texture, improved digestibility, and enhanced nutritional quality [1]. Pulses have gained increasing attention as raw materials for tempeh production due to their abundant protein, starch, dietary fiber, and other bioactive components that confer potential health benefits [2]. Conventional tempeh processing relies on dehulled seeds to facilitate water absorption, gas exchange, and fungal colonization. While dehulling improves fermentation efficiency, it removes the seed coat, a major reservoir of dietary fiber and related nutrients, thereby compromising the nutritional integrity of the final product [3]. Beyond this nutritional loss, the dehulling process itself is cumbersome and resource-intensive, which detracts from the sustainability of tempeh production. Given that international dietary guidelines consistently promote the consumption of whole pulses to maximize their health advantages [4], there is a compelling rationale for developing tempeh from whole, unrefined pulse seeds.

Black kidney bean (*Phaseolus vulgaris* L.) is particularly suitable for whole-bean applications owing to its high protein content, low fat level, and strong antioxidant activity [5]. However, whole-bean tempeh has received little research attention. The primary bottleneck is the seed coat, which constitutes a dense and impervious barrier due to its phenolic-rich composition and suberin-impregnated palisade cells [6]. This structure restricts water penetration, gas exchange, and microbial access to endosperm nutrients during fermentation [7]. Paradoxically, the same seed coat is a valuable source of dietary fiber, especially in pigmented varieties, and its removal inevitably diminishes the nutritional quality of the product [3]. This dual challenge, namely nutritional loss upon dehulling versus fermentation inefficiency with the intact coat, underlies the technical difficulty of developing whole-bean tempeh. Given that the intact seed coat restricts water penetration, gas exchange, and microbial access, pretreatment of the whole bean is a prerequisite for efficient tempeh fermentation, this study therefore focuses on the pretreatment.

Ultrasound has emerged as a promising non-thermal pretreatment technology in food processing, primarily due to its cavitation-induced mechanical effects [8], [9], [10], [11]. Among various non-thermal processing technologies, ultrasound is particularly favored for its simple equipment setup, ease of operation, and effectiveness in facilitating the release of bioactive compounds from plant matrices [12]. A recent study has shown that ultrasound treatment enhances the bioaccessibility of flavonoids and minerals in tempeh prepared from dehulled soybean [13]. More importantly, ultrasound can disrupt the compact structure of seed coats, thereby improving mass transfer and accelerating hydration and extraction processes. For instance, ultrasound-induced cracking on soybean seed coats accelerates water absorption and shortens sprouting time, while microporous channels created on mung bean epidermis directly promote water uptake [14], [15], [16]. These findings suggest that ultrasound may offer an effective strategy to enable successful fermentation of whole-bean tempeh, preserving its nutritional integrity while eliminating the dehulling step, thus reducing processing waste and enhancing the sustainability of the process.

Against this background, this study investigated the effects of ultrasound pretreatment on improving whole black kidney bean fermentation by modulating seed coat structure, thereby influencing fungal colonization and subsequent nutrient transformation. Compared with previous studies on legumes, which mainly focused on soaking, germination, or extraction and reported similar ultrasound-induced seed coat disruption, the novelty of this study lies in the following aspects: (1) it is the first to apply ultrasound pretreatment to whole-bean tempeh fermentation without dehulling, thereby preserving the nutrient-rich seed coat; (2) it focuses on the balance between protein bioaccessibility and starch digestibility; and (3) it aims to identify the optimal ultrasound condition that balances structural modification, fermentation efficiency, and nutrient retention. The structural modifications of the seed coat were characterized through changes in microstructure, hydration properties, and texture. Fermentation performance was evaluated by monitoring mycelial development and key hydrolytic enzyme activities. Nutritional conversions, including protein degradation and starch utilization, were analyzed to assess the balance between substrate breakdown and nutrient retention. Finally, *in vitro* digestion models were employed to evaluate the nutritional equilibrium of the final product, with particular emphasis on the trade-off between protein bioaccessibility and starch digestibility. This study provides new insights into the potential of ultrasound to upgrade the overall quality of whole-bean tempeh while retaining the seed coat and its associated nutritional benefits.

## 2 Materials and methods

### 2.1 Materials

Black kidney beans (*Phaseolus vulgaris* L.) were obtained from Heilongjiang Province, P. R. China.

Prestained protein marker (10–250 kDa) was purchased from Sangon Biotech (Shanghai, P. R. China). All other chemicals and reagents were of analytical grade.

The *Rhizopus oligosporus* RT-3 (Accession Number: M 20231023) was isolated from tempeh, a conventional fermented soy food of Indonesia. This strain was preserved from the China Center for Type Culture Collection (CCTCC, Wuhan University, P. R. China).

### 2.2 Ultrasound treatment

Ultrasonic treatment was performed using an ultrasonic processor (650 W, 20–25 kHz, HUXI, Shanghai, P. R. China) equipped with a 6 mm probe. Black kidney beans soaked for 15 h were treated at 60% of maximum power output for 10, 20 and 30 min under a 3‑seconds‑on/1‑second‑off pulse regime. Based on these settings, the total energy inputs were 175.5, 351.0, and 526.5 kJ for 10, 20, and 30 min, respectively. With a sample mass of 180 g (wet weight), the corresponding energy densities were 0.975, 1.95, and 2.925 kJ/g. The ultrasound settings were optimized to bring about gentle rearrangements of the interior framework, while avoiding complete gelling and aggressive polymer scission. During the whole process, samples were placed in an ice bath to prevent overheating, and the temperature was monitored to ensure it did not exceed 37 ℃. The control group (NC) was subjected to the same conditions without ultrasonic treatment. Ultrasound‑treated samples were labeled as U‑10, U‑20 and U‑30.

### 2.3 Seed coat permeability

#### 2.3.1 Cryo‑scanning electron microscopy (Cryo‑SEM)

The microstructure of black kidney bean seed coats before and after ultrasonic treatment was examined using Cryo‑SEM (SU8600, Hitachi, Tokyo, Japan). Samples were secured on a cryo‑sample holder, vitrified by rapidly plunging into liquid nitrogen, and a fine gold coating was then applied by cold sputtering. Images were acquired at 20.0  kV in a high‑vacuum environment. Pore area fraction on the seed coat surface was quantified using ImageJ platform (National Institutes of Health, Bethesda, MD, USA).

#### 2.3.2 Moisture content

The moisture content of the samples was determined by oven drying to constant weight.

(1) $$X=\frac{m_{1}-m_{2}}{m_{1}-m_{3}}\times 100$$

*X* (g/100  g): moisture content; *m_1_* (g): mass of weighing bottle and sample before drying; *m_2_* (g): mass of weighing bottle and sample after drying; *m_3_* (g): mass of weighing bottle.

#### 2.3.3 Water absorption and water absorption rate of seeds

Fifty seeds were placed in a conical flask containing 100 mL distilled water and subjected to ultrasonic treatment according to the conditions described in Section 2.2. At ten‑minute intervals, the seeds were removed, surface water was blotted dry, and the weight was recorded using an analytical balance. Water absorption percentage and absorption rate were calculated using the following equations [15].

Water absorption percentage:

(2) $$W_{P}\left(\%\right)=\frac{W_{n}-W_{0}}{W_{0}}\;$$

Water absorbing rate:

(3) $$W_{r}\left(g/min\right)=\frac{W_{n}-W_{n-1}}{t}\;$$

*W_0_*: initial weight of fifty seeds, *W_n_*: the seed mass measured at soaking time n, *W_n-1_*: the mass of the seeds at the previous time points of a certain time points of immersion, *t*: the interval between successive measurements, which was set to 10 min.

### 2.4 Texture properties

Texture properties of seeds were measured using a TMS‑PRO texture analyzer (Food Technology Corporation, Sterling, VA, USA) equipped with a 1000  N load cell and a cylindrical flat‑head aluminum probe. The probe compressed the cotyledon to 50% of its original height at a constant speed of 1  mm/s. Hardness, chewiness, springiness, gumminess and adhesiveness were recorded. At least ten replicates were performed for each sample.

### 2.5 Fourier transform infrared (FT‑IR) spectroscopy

FT‑IR spectra were performed in the region of 4000–500  cm^−1^ using a Nicolet Summit X spectrometer (Thermo Fisher Scientific, Waltham, MA, USA). A single seed coat was placed directly on the diamond crystal and clamped. Prior to sample introduction, the background was measured using the empty crystal. Spectra were collected in absorption mode by averaging 64 scans at 2  cm^−1^ intervals.

### 2.6 Preparation of tempeh

Black kidney beans (either with the dehulled kidney bean tempeh, DT; whole-kidney bean tempeh, WT; or ultrasound‑treated, WT-U10, WT-U20, WT-U30) were cooked for 20 min, then cooled to room temperature. Food‑grade lactic acid (1%, w/w) and starter culture (2%, w/w) were added and mixed thoroughly. Fermentation was carried out at 37 °C in an incubator, with slight modifications to the method of Sun et al. [1].

### 2.7 Observation of the integrity of cotyledon cells

#### 2.7.1 Optical microscopy

The isolation of cotyledonary cells was performed according to a published protocol with minor modifications [17]. The samples were mashed and filtered through 100-mesh and 300-mesh sieves. The portion held back on the finer screen, which contained these cells, was collected. The recovered cotyledonary cells were re-suspended in distilled water and examined under an optical microscope (ECLIPSE E100, Nikon, Tokyo, Japan).

#### 2.7.2 Confocal laser scanning microscopy (CLSM)

Cotyledon cells were isolated as described in Section 2.7.1 and pre-stained with Calcofluor White, Rhodamine B, and fluorescein isothiocyanate (FITC) to label cell walls, proteins, and starch, respectively, as previously described [1]. The stained samples were visualized using a Leica laser scanning confocal microscope (Leica Microsystems, Wetzlar, Germany). Laser excitation was performed at 405, 488, and 543 nm. Images were acquired using a 10× objective.

### 2.8 Determination of ergosterol

A rotary evaporator (Hei-V A, Heidolph, Schwabach, Germany) was employed to concentrate the extract, and ergosterol was then isolated following a previously described protocol [18]. Quantification was performed by high‑performance liquid chromatography (HPLC, LC-20A, Shimadzu, Japan) equipped with a Zorbax SB‑C18 column (4.6  mm × 250  mm, 5  μm; Santa Clara, CA, USA). The injection volume was 20  μL. Elution was carried out with 100% methanol at 1.5  mL/min, with the column temperature at 25 °C. Absorbance was monitored at 282  nm.

### 2.9 Estimation of enzyme activity

Protease activity was estimated with a slight modification of a previously described protocol [19]. α‑Amylase and β‑glucosidase activities were measured following Bhanja et al. [20]. Endo‑β‑glucanase activity was assayed using carboxymethyl cellulose sodium as the substrate, whereas exo‑β‑glucanase activity was determined using microcrystalline cellulose as the substrate.

### 2.10 Analysis of protein

#### 2.10.1 Determination of the molecular weight distribution

Samples were homogenized, adjusted to pH 9.0 with sodium hydroxide, and extracted in a water bath at 50 °C for 1 h with shaking at 120 rpm. The mixture was centrifuged at 11,100*×g* for 20 min at 4 °C. The extracted proteins were filtered through a 0.45 μm pore size. Size-exclusion HPLC (SEC-HPLC) was used to determine the molecular weight distribution of the proteins, according to a previously described method with modifications [21]. Separation was performed on a TSKgel G3000SWXL column (7.8 mm ID × 30 cm L). The mobile phase consisted of 0.1 mol L^−1^ sodium phosphate buffer, 0.1 mol L^−1^ sodium sulfate, and 0.05% sodium azide, at a flow rate of 1 mL min^−1^. The injection volume was approximately 50 μL, and eluents were detected at 280 nm.

#### 2.10.2 SDS-PAGE

Protein extraction was performed as described in Section 2.10.1. The resulting supernatant was combined with 4 × protein loading buffer (3:1, v/v) and boiled for 5  min, then centrifuged again for 2  min. SDS-PAGE was performed according to a previously described method with modifications [22]. Electrophoresis was performed on a polyacrylamide gel at a constant voltage of 80  V until the dye front reached the bottom of the gel. A pre‑stained protein marker (10–250  kDa) was used as a standard. Protein band analysis was conducted using Quantity One software (Bio‑Rad Laboratories, Inc., Hercules, CA, USA).

#### 2.10.3 Peptide content

Samples were homogenized with distilled water at a 1:5 (w/v) ratio and centrifuged. An equal volume of 10% TCA was added to the supernatant, and the mixture was allowed to stand for 2  h before being centrifuged at 2290 *× g* for 15 min at 4 °C. The resulting supernatant was used for the assay. A reagent mixture containing 100  mM sodium tetraborate, 20% sodium dodecyl sulfate, o‑phthaldialdehyde and β‑mercaptoethanol was prepared and kept in the dark. The assay supernatant was mixed with the reagent and absorbance was measured at 340  nm according to Rui et al. with modifications [23].

### 2.11 Analysis of starch and starch degradation products

#### 2.11.1 Total starch content

The methodology was adopted from a previous study with slight modification [24]. Samples were sequentially dispersed in ethanol and dimethyl sulfoxide, vortexed, and boiled for 5  min to disrupt resistant starch. After adding thermostable α‑amylase, the mixture was boiled for another 6  min. Following cooling, sodium acetate buffer and amyloglucosidase were added and incubated at 50 °C for 30  min. The supernatant was collected by centrifugation and mixed with GOPOD reagent (glucose oxidase, peroxidase, and 4‑aminoantipyrine). After incubation at 50 °C for 20  min, absorbance was measured at 510  nm.

#### 2.11.2 Reducing sugar content

The reducing sugar content was determined using the 3,5-dinitrosalicylic acid (DNS) method with minor modifications [25]. The fermented sample was homogenized with distilled water and incubated in a shaking water bath at 50 °C for 30  min, then centrifuged at 2775 × *g* for 15  min at 4 °C. The supernatant was combined with DNS reagent, heated in a boiling water bath for 5  min, and immediately cooled in an ice bath. Absorbance was measured at 540  nm.

#### 2.11.3 Iodine blue value

The iodine blue value was determined according to a previous study with slight modifications [26]. The sample was added to distilled water, placed in a water bath and agitated at 120  rpm for 5  min. Standard iodine solution was added, and absorbance at 660  nm was subsequently measured.

### 2.12 In vitro simulated digestion

#### 2.12.1 The process of *in vitro* gastrointestinal simulated digestion

*In vitro* digestion was performed according to the INFOGEST static protocol [27]. Oral phase: samples were mixed with simulated salivary fluid and α‑amylase (final activity 75 U/mL), and incubated at 37 °C for 2  min. Gastric phase: simulated gastric fluid, pepsin solution (final activity 2000 U/mL) and CaCl_2_ were added. The pH was adjusted to 3.0 with HCl, and incubation was carried out at 37 °C with shaking at 55  rpm for 120  min. Intestinal phase: Simulated intestinal fluid, pancreatin (final activity 100 U/mL), CaCl_2_ and bile salts (10 mM) were added. The pH was adjusted to 7.0 with NaOH, and incubation was performed at 37 °C with shaking at 180  rpm for 120  min. At the end of each phase, the enzymatic reactions were stopped by heating in boiling water for 5  min. The samples were then centrifuged, and the supernatants stored at −20 °C.

#### 2.12.2 Readily bioaccessible protein

Readily bioaccessible protein was determined according to Pälchen et al. [28]. Digested samples were mixed with 3.5% TCA, allowed to stand, and centrifuged at 10,000*×g* for 10 min at 4 °C. 40 μL of the supernatant was mixed with 200  μL OPA reagent, incubated in the dark for 2  min, and absorbance was measured at 340  nm.

(4) $$Readily\;bioaccessible\;protein\;\left(\%\right)=\frac{{NH}_{2\left(TCA\right)}-{NH}_{2\left(initial\right)}}{{NH}_{2\left(total\right)}-{NH}_{2\left(initial\right)}}\times 100$$

*NH_2(TCA)_* (mg/mL): the concentration of α-amino groups derived from TCA-hydrolyzed digestate; *NH_2(initial)_* (mg/mL): the level of α-amino groups pre-existing in the undigested sample; *NH_2(total)_* (mg/mL): the concentration of α-amino groups after hydrolysis with 6  N HCl at 110 °C for 16  h.

#### 2.12.3 Starch digestibility and eGI

The method was adapted from previous studies [29]. Maltose released during digestion was quantified using the 4‑hydroxybenzaldehyde hydrazine (PAHBAH) method. The supernatant was mixed with 0.5% (w/v) PAHBAH, heated in a water bath at 100℃ and then cooled in an ice bath. Absorbance was measured at 410  nm.

(5) $$Maltose\;equivalent\;release\;\left(\%\right)=\frac{maltose\;equivalent\;\times \;0.95}{total\;starch\;content}\times 100$$

Starch hydrolysis curves were plotted using Origin software, and the area under the curve (AUC) was calculated. The hydrolysis index (HI) was obtained by dividing the AUC of the sample by that of white bread:

(6) $$HI=\frac{\mathrm{AU}C_{\mathrm{sample}}}{\mathrm{AU}C_{\mathrm{whitebread}}}\times 100$$

The eGI value was then calculated according to Granfeldt et al. [30]:

(7) eGI=0.862×HI+8.198

### 2.13 Statistical analysis

All determinations were conducted in triplicate with independently processed samples, and values are expressed as the mean ± standard deviation (SD). For data with only one factor, one-way ANOVA followed by Duncan’s multiple range test was used. For data involving both treatment and fermentation time, two-way ANOVA was used to evaluate the main effects of treatment and time as well as their interaction. When the interaction was significant, treatments were compared at each fermentation time point using Duncan’s multiple range test. Statistical significance was set at *p* < 0.05. All analyses were performed using SPSS software (IBM Corp., NY, USA). Graphs were prepared with Origin 2024 (Stat-Ease Inc., MN, USA).

## 3 Results and discussion

### 3.1 Effect of ultrasound treatment on black kidney bean seed coat structure

Fig. 1A illustrates the experimental workflow for characterizing black kidney beans and their seed coats. Fig. 1B shows Cryo-SEM images of the seed coat surface. The untreated sample (NC) exhibited a continuous, compact, and relatively smooth surface with no macroscopic defects. In contrast, all ultrasound-treated samples (U-10, U-20, and U-30) displayed clear signs of surface disruption. After 10 min of treatment, initial micro-cracks and localized exfoliation appeared. Extending the treatment to 20 min and 30 min caused more severe morphological changes, including wide and deep grooves, large-scale peeling, and markedly increased roughness. SEM is effective in capturing such processing-induced microstructural changes on food surfaces [31]. These effects are attributed to cavitation-induced shockwaves and microjets, which disrupt physical cross-linking and intermolecular interactions within the epicuticular wax and cell wall polysaccharides, leading to irreversible mechanical erosion and fracturing [32]. The ultrasonically treated seed coat thus lost much of its intact physical barrier, which in turn improved its wettability and permeability.

**Fig. 1:**
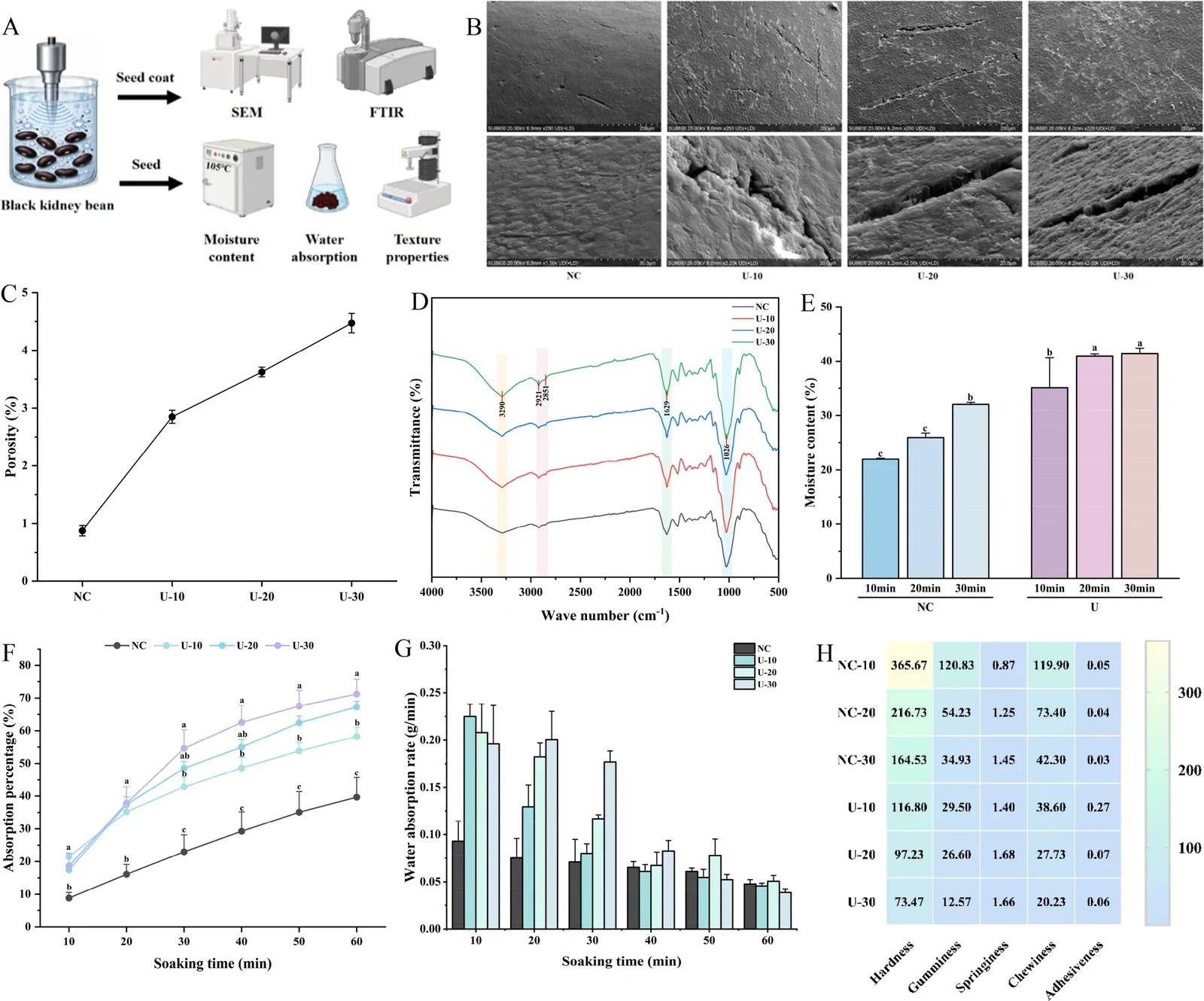
Characterization of black kidney beans and their seed coats under different ultrasound treatment times (untreated, ultrasound for 10 min, 20 min, and 30 min). (A) Schematic workflow of the study. (B) Cryo-SEM images of the seed coat surface. (C) Porosity of seed coats. (D) FTIR spectra of seed coats. (E) Moisture content. (F) Water absorption percentage. (G) Water absorption rate. (H) Textural parameters. Data are presented as mean ± SD. One-way ANOVA followed by Duncan's multiple range test was used for pairwise comparisons. Different letters (a, b, c) denote statistically significant differences (*p* < 0.05). These results indicate that ultrasound treatment alters the physical and structural properties of black kidney bean seed coats.

Quantitative analysis of seed coat porosity based on the Cryo-SEM images (Fig. 1C) confirmed these structural changes. The NC had a dense microstructure with a basal porosity of 0.9%. Porosity increased significantly with ultrasound duration, reaching approximately 2.9%, 3.6%, and 4.5% after 10, 20, and 30 min, respectively (*p* < 0.05). The most pronounced increase occurred within the initial 10 min, while the rate of increase slowed notably between 20 and 30 min. This trend suggests that the initial rapid rise resulted from the sudden rupture of the dense outer cuticle and wax layers, whereas the later stage mainly involved the widening and deepening of existing micro-cracks rather than the generation of new fractures. Study has shown that porosity is one of the key structural parameters governing water migration and solute permeability in food matrices [33].

FTIR spectra of the seed coats (Fig. 1D) displayed the typical features of polysaccharide-rich biomaterials. Compared with the NC, the ultrasound-treated samples generally showed deeper absorption valleys at characteristic wavenumbers. At 3290 cm^−1^ (O-H stretching) and 2921/2851 cm^−1^ (aliphatic C-H stretching), the absorption valleys deepened progressively from U-10 to U-30, indicating that prolonged ultrasound exposure increasingly disrupted the hydrophobic surface barrier and exposed more hydroxyl groups. However, the spectral evolution at 1629 cm^−1^ (aromatic or conjugated carbonyl vibrations) and 1026 cm^−1^ (C-O/C-O-C stretching of polysaccharides) followed a different pattern. The NC showed broad and relatively shallow valleys. After 10 min of treatment, these valleys became narrower and deeper, with the sharpest features observed at U-20. At U-30, however, the valleys became noticeably broader and shallower than those at U-20. This reversal suggests that while 20 min of ultrasound maximized the exposure of these functional groups, excessive cavitation at 30 min may have led to a different structural state of the polysaccharide chains. Overall, the spectral data indicate that ultrasound primarily caused structural reorganization rather than extensive chemical degradation, increasing the accessibility of hydrophilic groups and weakening the surface hydrophobic barrier [34].

### 3.2 Effect of ultrasound treatment on moisture uptake of black kidney beans

Fig. 1E presents the moisture content of NC and ultrasound-treated samples over time. The ultrasound-treated group exhibited significantly higher moisture at each measured time point (*p* < 0.05). Their moisture content increased rapidly from approximately 35% at 10 min to 41% at 20 min, after which it plateaued. In contrast, the NC showed only a gradual increase from 22% at 10 min to 26% at 20 min and remained consistently lower throughout. These observations are consistent with Sonowal et al. [35], who reported that ultrasound treatment significantly accelerated water uptake in kidney beans compared with conventional soaking.

Fig. 1F and G further illustrate the effects of ultrasound treatment on water absorption percentage and water absorption rate over 60 min of soaking. Ultrasound treatment significantly enhanced water uptake, with absorption capacity reaching 1.5 to 2.4 times that of the NC throughout the soaking period. At the 10 and 20 min of the initial soaking phase, all ultrasound-treated samples showed comparable absorption levels, suggesting that the seed coat barrier remained largely intact at the very early stage. As soaking progressed, clear differences emerged among groups. By the end of soaking, U-20 and U-30 reached final water absorption values of 67% and 71%, respectively, with no significant difference between them. Both values were substantially higher than that of the NC (40%), indicating that ultrasound promoted water diffusion into the bean matrix.

As shown in Fig. 1G, the water absorption rate declined gradually over time in all samples. The decline was steeper in the ultrasound-treated groups, whereas the NC exhibited a more moderate pattern. At 10 and 20 min, the water absorption rates of the ultrasound-treated groups were 1.7–2.7 times those of the NC. At 30 min, U-20 (0.12 ± 0.01 g/min) and U-30 (0.18 ± 0.01 g/min) still maintained relatively high rates, while U-10 (0.08 ± 0.01 g/min) was comparable to the NC (0.07 ± 0.02 g/min). Between 40 and 60 min, no significant differences in water absorption rate were observed among any of the groups. These results demonstrate that ultrasound exerts a pronounced effect during the early stage of water uptake by significantly enhancing the permeability of the black kidney bean seed coat. Similar results were reported by Chen et al. [15], who confirmed that ultrasound can improve soybean seed coat permeability.

### 3.3 Texture properties

The texture properties of black kidney beans, including hardness, gumminess, springiness, chewiness, and adhesiveness, are summarized in Fig. 1H. Overall, ultrasound-treated samples exhibited a significantly softer and less compact texture compared with the NC.

Differences in hardness emerged as early as 10 min of treatment. The hardness of U-10 was 116.80 ± 8.25 N, approximately 68% lower than that of the NC at the same time point (365.67 ± 4.87 N), indicating that ultrasound treatment accelerated tissue softening during the initial hydration stage. At U-20 and U-30, the hardness further decreased to 97.23 ± 2.78 N and 73.47 ± 2.06 N, respectively, representing reductions of approximately 55% relative to the corresponding NC. Similar trends were observed for gumminess and chewiness, both of which are derived from hardness and cohesiveness. The gumminess and chewiness values in the ultrasound-treated groups were significantly lower, accounting for 24%-50% and 32%-48% of the NC values, respectively.

Notably, the ultrasound-induced changes in springiness and adhesiveness stabilized after 20 min of treatment. Springiness increased from 1.40 ± 0.12 mm at U-10 to 1.68 ± 0.01 mm at U-20, with no further change at U-30 (1.66 ± 0.08 mm). In contrast, the NC groups exhibited lower springiness values ranging from 0.87 ± 0.02 to 1.45 ± 0.02 mm. Likewise, the adhesiveness of the ultrasound-treated samples declined from 0.27 ± 0.01 N·mm at U-10 to 0.07 ± 0.01 N·mm at U-20, and remained essentially unchanged at U-30. Although the ultrasound-treated groups consistently showed slightly higher adhesiveness than the NC groups (0.03–0.05 N·mm), the absolute values across all samples were extremely low throughout the entire timeframe. Thus, ultrasound treatment did not meaningfully affect the adhesiveness of the beans in practical terms.

The textural properties of foods are closely related to their microstructure, and non-uniform structural changes can influence textural performance [36]. These textural changes were consistent with the increased seed coat porosity and enhanced water absorption capacity observed in the ultrasound-treated samples (3.1, 3.2). Collectively, the results demonstrate that ultrasound treatment effectively reduces resistance to compression and fracture [37], yielding a softer yet more elastic texture, with near-optimal properties achieved within 20 min.

### 3.4 Fermentation characteristics of black kidney bean

#### 3.4.1 Macroscopic and microscopic morphology of tempeh and cotyledon cells

Fig. 2A shows the visual appearance of dehulled tempeh (DT), whole-bean tempeh (WT), and ultrasound-treated tempeh (WT-U10, WT-U20, WT-U30) at 0 h and 32 h of fermentation. At 32 h, the DT exhibited yellowish mycelium with visible shrinkage, indicating rapid substrate depletion and early entry into the decline phase. WT was predominantly covered with mature black spores, reflecting incomplete surface colonization and limited nutrient utilization. In contrast, all ultrasound-treated samples displayed more complete white mycelial coverage. Among them, WT-U20 showed the most superior colonization, characterized by uniform white mycelium, a compact cross-section fully penetrated by internal mycelium, and no mature sporulation. These observations suggest that moderate ultrasound treatment created a favorable fermentation microenvironment, with 20 min likely providing an optimal balance between improved mass transfer and substrate stability.

**Fig. 2:**
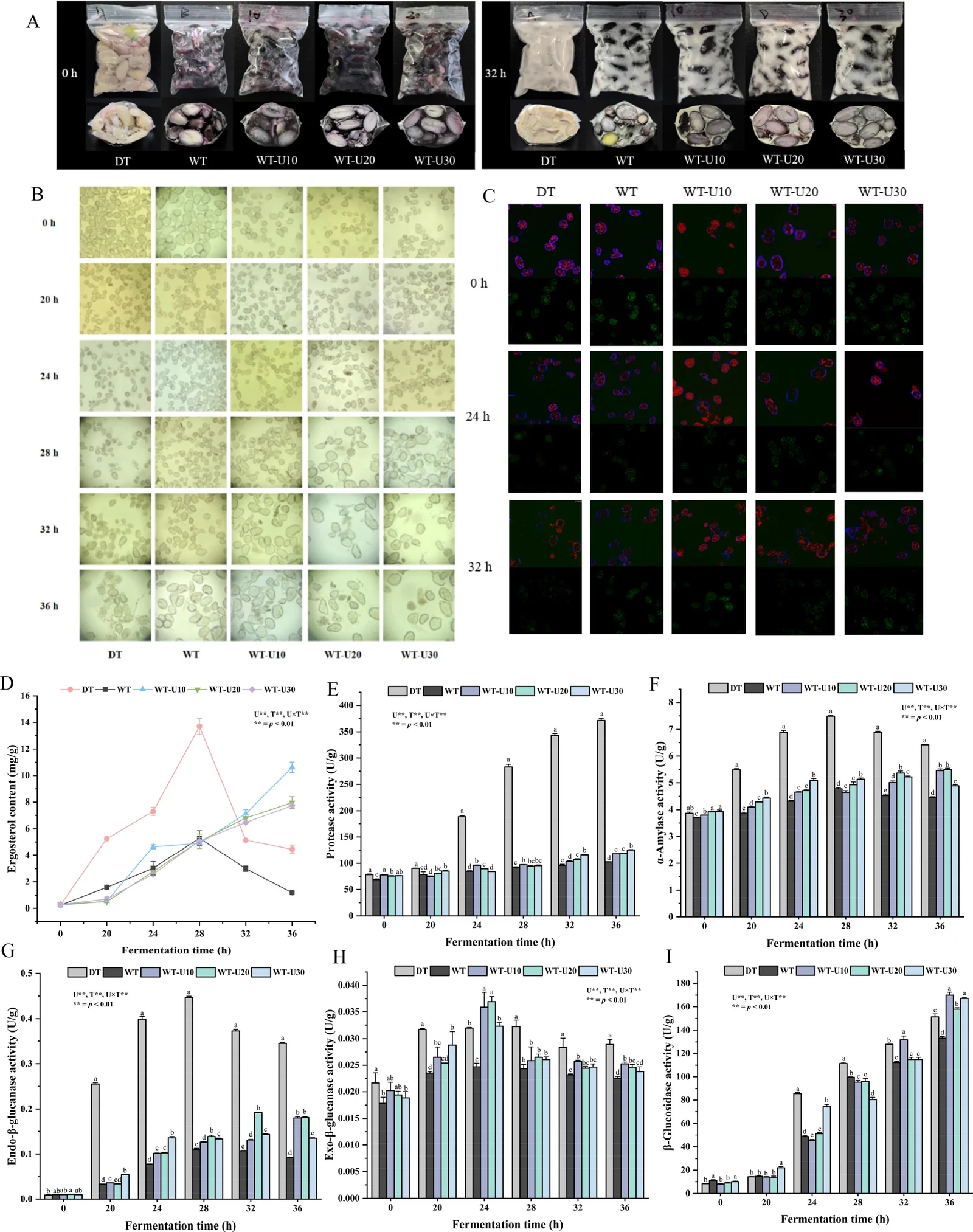
Microbial growth and fermentation efficacy of black kidney beans under different treatment conditions. (A) Visual appearance of black kidney beans at 0 h and 32 h of fermentation. (B) Optical microscopy images of cotyledon cells during fermentation. (C) CLSM images of cotyledon cells with specific fluorescent labelling of cell walls (blue), proteins (red), and starch (green). (D) Ergosterol content. Enzyme activities: (E) protease, (F) α-amylase, (G) *endo*-β-glucanase, (H) *exo*-β-glucanase, and (I) β-glucosidase. Data are presented as mean ± SD. Different letters indicate significant differences among treatments at the same fermentation time (*p* < 0.05). U, treatment effect; T, fermentation time effect; U × T, interaction effect; ** means significant difference at *p* < 0.01.

Optical microscopy (Fig. 2B) revealed that cotyledon cells in all groups gradually transitioned from relatively round and intact structures at early fermentation stages to irregular shapes at later stages, indicating progressive microstructural disassembly. However, the timing and extent of these changes varied among treatments. In the WT, cotyledon cells largely retained a relatively rounded morphology. In contrast, the DT exhibited more evident structural alteration after 24 h of fermentation, suggesting that dehulling facilitated fermentation access to cotyledon tissues. The ultrasound-treated groups exhibited earlier and more evident microstructural changes than WT. In particular, WT-U20 and WT-U30 already showed visible morphological alterations at 28 h, which became more pronounced at 32 and 36 h with clearly irregular cell contours. For WT-U10, the extracellular region appeared blurred at 32 h, accompanied by leakage of intracellular material. Overall, ultrasound treatment effectively accelerated cotyledon cell disassembly during fermentation even without seed coat removal, an effect attributed to improved microbial accessibility to the cotyledon matrix, thus partially substituting for the need for dehulling.

To further verify the structural changes in cotyledon cells, CLSM was performed with specific fluorescent labelling of cell wall components (blue), proteins (red), and starch (green) (Fig. 2C). At 0 h, all samples exhibited intact cell structures with clear cell wall boundaries, and both protein and starch signals were confined within the cells. After 24 h of fermentation, DT showed pronounced cell wall disruption, with diffused protein signals and reduced starch granules. WT retained relatively intact cell structures, whereas ultrasound-treated groups, especially WT-U20, exhibited earlier and more severe cell wall degradation, accompanied by the release of proteins and starch. By 32 h, the cell structures in DT were largely disintegrated, and starch was almost depleted. WT still contained a considerable number of intact cells, while WT-U20 showed the most extensive structural disruption among the ultrasound groups, closely resembling DT. WT-U10 and WT-U30 also showed enhanced degradation compared with WT, but to a lesser extent than WT-U20. These CLSM observations provide direct evidence that ultrasound pretreatment, particularly for 20 min, promotes the degradation of cotyledon cell walls and the release of intracellular components during fermentation.

#### 3.4.2 Growth of *R. oligosporus* on black kidney bean

Ergosterol, used as an indicator of filamentous fungal growth, is shown in Fig. 2D. Two-way ANOVA showed that treatment, fermentation time, and their interaction significantly affected ergosterol content (*p* < 0.01). During the first 28 h of fermentation, ergosterol increased gradually across all samples, with DT reaching the highest value of 13.70 ± 0.61 mg/g. This rapid accumulation in DT reflected the removal of the seed coat, which provided unhindered nutrient access at the cost of accelerated substrate depletion. After 28 h, ergosterol began to decline in DT and WT, but continued to increase in all ultrasound-treated samples, eventually exceeding the corresponding DT and WT values at 36 h. At this time point, the ergosterol content of WT-U10 reached 10.63 ± 0.39 mg/g, approximately 9.0-fold higher than that of WT and 2.4-fold higher than that of DT. These results demonstrate that ultrasound pretreatment prolonged the vegetative growth phase of *R. oligosporus*, whereas fungal growth in the non-ultrasound-treated groups had already entered the decline phase.

The activities of five key hydrolytic enzymes during fermentation are compared in Fig. 2E-I. For all five enzymes, treatment, time, and their interaction were significant (*p* < 0.01). Ultrasound treatment significantly enhanced hydrolase activities during the mid-to-late fermentation stages, with differences among groups widening over time. Protease activity at 36 h reached 125.14 ± 0.94 U/g in WT-U30 (1.3-fold higher than WT), while WT-U10 and WT-U20 reached 118.17 ± 0.19 and 118.38 ± 0.27 U/g, respectively (both 1.2-fold higher than WT). α-Amylase activity showed an even more pronounced response. At 36 h, WT-U10 and WT-U20 reached 5.46 ± 0.06 and 5.50 ± 0.05 U/g, respectively, compared with 4.45 ± 0.04 U/g for WT, though both remained below DT (6.42 ± 0.01 U/g). This suggests that ultrasound-induced physical disruption partially improved starch exposure but could not fully replicate the effect of complete barrier removal.

For cellulolytic enzymes, *endo*-β-glucanase activity in ultrasound-treated samples was 1.2 to 2.0-fold higher than that in WT after 20 h of fermentation. Exo-β-glucanase activity was overall 1.1 to 1.5-fold higher than that of WT, and at 24 h, the values in WT-U10 and WT-U20 exceeded those of DT by 1.1 to 1.2-fold. β-Glucosidase activity was particularly enhanced by ultrasound pretreatment. At 32 h and 36 h, the activities in WT-U10 (131.76 ± 3.18 and 169.99 ± 2.52 U/g, respectively) were significantly higher than those of both DT and WT, reaching approximately 1.1-fold higher than DT and 1.3-fold higher than WT. Given that these enzymes are directly involved in cell wall polysaccharide depolymerization and glycosidic bond cleavage, their enhancement indicates a greater capacity for disrupting cotyledon cell wall integrity and releasing intracellular components [38]. Previous studies have reported ultrasound-induced increases in individual enzyme activities in simple matrices such as soybean meal and okara, including a 16.58% increase in protease activity and β-glucosidase activity up to 15.50 U/mL [39], [40]. In contrast, the whole-bean tempeh system with an intact seed coat involves the synergistic action of multiple hydrolytic enzymes, and the increases observed were greater than those reported in these simple matrices.

### 3.5 Nutritional transformation during fermentation

Ultrasound pretreatment profoundly altered the pattern of nutrient transformation during fermentation, encompassing protein degradation and starch utilization.

Regarding protein degradation, SEC was used to evaluate changes in protein molecular weight distribution during fermentation (Fig. 3A). At 0 h, all samples were dominated by high-molecular-weight species (>500 kDa and 100–500 kDa). As fermentation proceeded, all groups showed a progressive shift from high-molecular-weight to low-molecular-weight peptides (<10 kDa and 10–50 kDa). At 32–36 h, WT still contained a considerable fraction of > 500 kDa aggregates, whereas ultrasound-treated groups, especially WT-U20, exhibited a more pronounced reduction in high-molecular-weight species and a corresponding increase in low-molecular-weight peptides. At 36 h, the molecular weight distribution of WT-U20 was closest to that of DT, with the > 500 kDa fraction even lower than that of DT. These results indicate that ultrasound pretreatment, particularly for 20 min, promotes protein degradation and reduces aggregation.

**Fig. 3:**
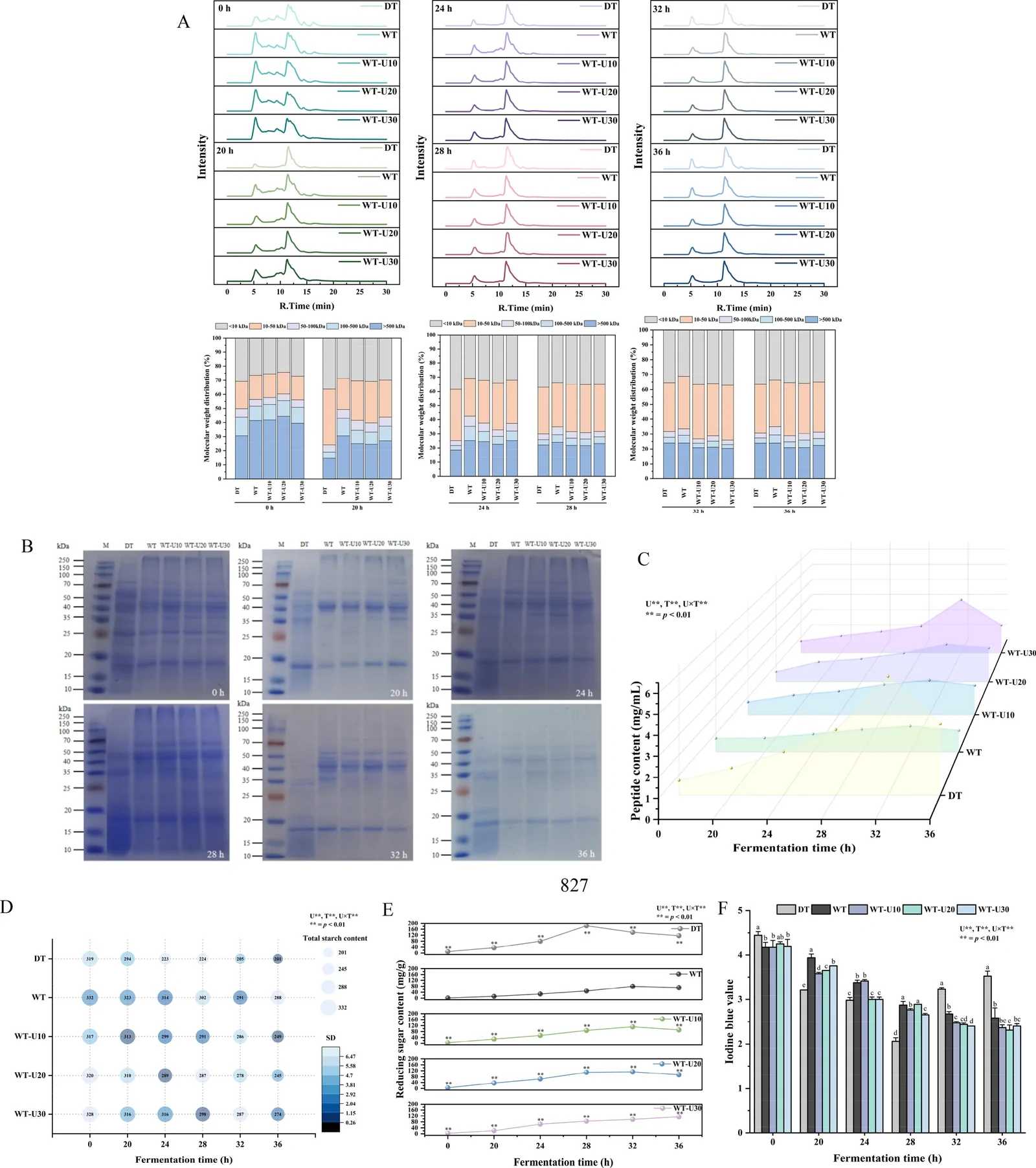
Nutritional transformation of black kidney beans during fermentation. (A) SEC profiles and molecular weight distribution percentages. (B) SDS-PAGE profiles; M indicates the molecular weight marker. (C) Peptide content. (D) Total starch content. (E) Reducing sugar content. (F) Iodine blue value. Data are presented as mean ± SD. Different letters indicate significant differences among treatments at the same fermentation time (*p* < 0.05). U, treatment effect; T, fermentation time effect; U × T, interaction effect; ** means significant difference at *p* < 0.01.

Consistent with these SEC findings, SDS-PAGE analysis (Fig. 3B) revealed that ultrasound treatment significantly enhanced breakdown of major storage proteins (convicilin at 70 kDa, vicilin subunits at 51, 35, 25 kDa, and legumin acidic/basic subunits at 40/18 kDa) [41]. In WT, bands around 70–72 kDa persisted through 24 h, indicating limited enzymatic access. At 32 h, WT-U10 showed weaker legumin and vicilin bands than WT. By 36 h, WT-U20 exhibited the faintest residual high-molecular-weight bands among ultrasound groups, approaching the degradation profile of DT. For peptide content (Fig. 3C), a significant treatment × time interaction was observed (*p* < 0.01). Throughout fermentation, ultrasound-treated groups maintained 1.4- to 2.5-fold higher peptide levels than WT, with WT-U20 showing the most sustained increase. WT-U20 displayed a sustained release profile between 20 and 28 h, and at 36 h maintained the highest peptide level among ultrasound groups (1.8-fold higher than WT), outperforming WT-U10 and WT-U30 in late-stage persistence. This may be attributed to ultrasound-induced protein unfolding that enhances enzyme accessibility [42]. Unlike the single-step ultrasound-assisted hydrolysis of legume protein isolates [43], peptide accumulation in whole-bean tempeh involves both ultrasound pretreatment and fungal protease activity during fermentation.

For starch metabolism (Fig. 3D-F), ultrasound treatment promoted starch hydrolysis while avoiding the excessive depletion observed in DT. WT-U20 showed the highest net starch consumption (75.22 mg/g), approximately 72% higher than WT but only 64% of DT. Its final residual starch was roughly 15% lower than WT yet 22% higher than DT, indicating a moderate hydrolysis profile. Reducing sugar content (Fig. 3F) in WT-U20 increased steadily during 20–32 h, reaching 112.88 mg/g at peak, second only to DT (139.44 mg/g) and significantly above WT (79.66 mg/g). Iodine blue values (Fig. 3G), reflecting amylose content, showed that WT-U20 reached the lowest value among ultrasound groups at 36 h (2.31), indicating effective degradation of ordered glucan regions without the retrogradation rebound observed in DT [44]. WT-U30 showed relatively lower reducing sugar accumulation than U10 and U20, possibly due to cavitation-induced oxidation of released sugars [45].

Collectively, ultrasound pretreatment enabled a balanced nutritional transformation profile distinct from both WT (limited substrate accessibility) and DT (excessive, uncontrolled degradation). WT-U20 consistently emerged as the optimal condition, achieving pronounced protein hydrolysis, and moderate starch utilization, while preserving sufficient substrate structure to sustain fungal metabolism throughout fermentation. This balance underlies the superior fermentation performance of ultrasound-pretreated whole-bean tempeh.

### 3.6 Digestive characteristics of fermented black kidney beans

#### 3.6.1 Readily bioaccessible protein

Fig. 4 (A1-A3) shows the readily bioaccessible protein across treatment groups during simulated gastrointestinal digestion. In all samples, bioaccessible protein increased progressively from the gastric phase (G-120) to the intestinal endpoint (I-300), reflecting gradual matrix disintegration and protein release.

**Fig. 4:**
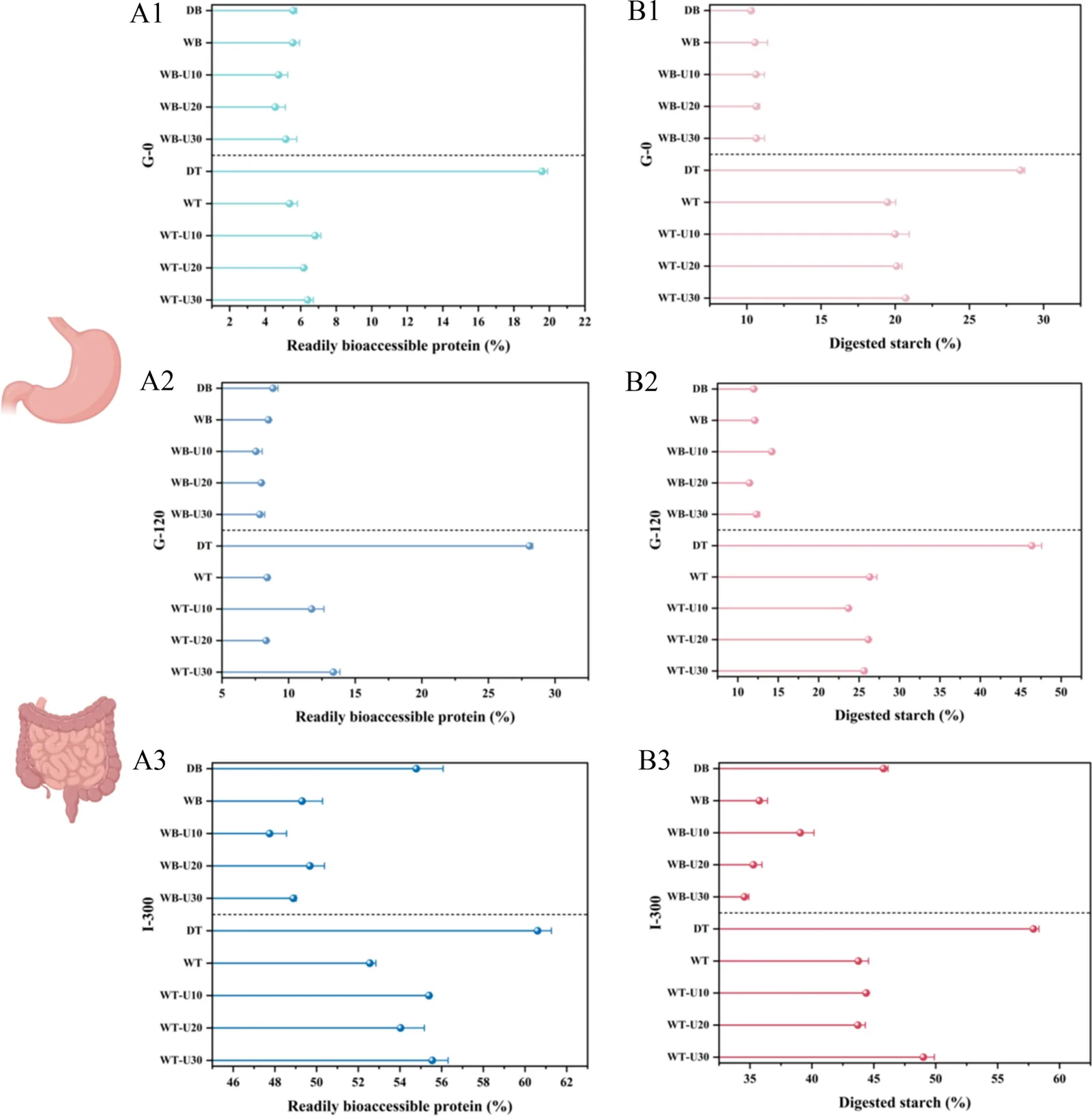
Digestive properties of black kidney bean samples at 0 h and 32 h of fermentation. (A1-A3) Readily bioaccessible protein content, and (B1-B3) starch digestibility, at the undigested stage, after gastric digestion, and after intestinal digestion, respectively. Data are presented as mean ± SD.

Ultrasound-treated tempeh consistently exhibited higher protein accessibility than WT at all digestion stages. During the gastric phase, ultrasound groups reached 12.08%-13.88%, representing a 30%-40% increase over WT (10.07%). At the intestinal endpoint, ultrasound-treated groups achieved 54.03%-55.55% protein bioaccessibility, surpassing WT and bridging approximately 37% of the gap between WT and DT. This result confirms that ultrasound effectively compromises the seed coat barrier, bringing the protein bioaccessibility of whole beans substantially closer to the dehulled tempeh [46]. Notably, WT-U20 reached about 92% of the DT level while preserving the whole-bean matrix, demonstrating its potential for applications requiring both improved nutritional accessibility and structural integrity.

#### 3.6.2 Starch digestibility and estimated glycemic index

Fig. 4 (B1-B3) presents the starch digestion profiles of different samples. While digestibility increased with digestion time across all groups, marked differences in hydrolysis rate and final extent were observed between treatments.

At the gastric endpoint (G-120), ultrasound-treated groups showed hydrolysis degrees (23.68%-26.14%) comparable to WT (26.32%), whereas DT reached 46.36%. This indicates that ultrasound did not trigger the massive starch release observed with dehulling. At the intestinal endpoint (I-300), DT reached 57.89%, while ultrasound groups ranged from 43.70% (WT-U20) to 49.00% (WT-U30), comparable to or slightly above WT (43.74%). These data demonstrate that the starch hydrolysis behavior of ultrasound-treated samples remained largely constrained by the whole-bean physical barrier, resembling the WT profile rather than the fully liberated dehulled state.

This distinct hydrolysis pattern reflects a dual role of ultrasound. The physical perturbation facilitates starch degradation during fermentation, yet may simultaneously induce molecular rearrangements that restrict enzyme accessibility during subsequent *in vitro* digestion [47], [48]. Consequently, the starch digestion behavior of ultrasound-treated tempeh results from a combination of structural opening and reorganization.

These differences translated into the hydrolysis index (HI) and estimated glycemic index (eGI) values (Table 1). Fermentation increased HI and eGI in all samples compared with unfermented beans. DT exhibited the highest eGI (67.24 ± 0.07), reflecting enhanced starch availability at the cost of glycemic control. In contrast, ultrasound-treated tempeh maintained substantially lower eGI values (WT-U10: 47.27 ± 0.52; WT-U20: 49.68 ± 0.24; WT-U30: 49.40 ± 0.05), comparable to WT (49.68 ± 0.24) and significantly below DT. This moderate eGI stems from the incomplete disruption of the seed coat barrier. Although ultrasound improved hydration and fermentation accessibility, the residual dietary fiber-rich outer layer preserved the structural integrity of the matrix, restricting rapid starch hydrolysis in a manner fundamentally different from complete dehulling. This contrasts with the findings of Pasumarthi and Manickavasagan [49], who reported that ultrasound-assisted isolation of pinto bean starch increased starch digestibility and eGI, as starch was directly exposed to cavitation in the absence of intact cell walls or seed coats.

**Table 1:** HI and eGI of non-fermented (0 h) and 32 h-fermented black kidney bean samples.

Sample	HI	eGI
DB	36.94 ± 0.27^h^	40.04 ± 0.23^h^
WB	31.48 ± 0.19^h^	35.33 ± 0.17^h^
WB-U10	34.05 ± 0.16^f^	37.55 ± 0.14^f^
WB-U20	31.96 ± 0.36^g^	35.75 ± 0.31^g^
WB-U30	32.55 ± 0.16^g^	36.26 ± 0.14^g^
DT	68.49 ± 0.08^a^	67.24 ± 0.07^a^
WT	47.20 ± 0.49^c^	48.89 ± 0.42^c^
WT-U10	45.33 ± 0.60^d^	47.27 ± 0.52^d^
WT-U20	48.12 ± 0.27^b^	49.68 ± 0.24^b^
WT-U30	47.80 ± 0.05^b^	49.40 ± 0.05^b^

Data are presented as mean ± SD. Values followed by different letters in the same column are significantly different (*p* < 0.05).

## 4 Conclusion

This study demonstrated that ultrasound pretreatment effectively modified the seed coat structure of black kidney beans, enabling successful whole-bean tempeh fermentation without dehulling. Ultrasound treatment increased seed coat porosity from 0.9% to 4.5%, enhanced water uptake and texture softening, and promoted fungal colonization and hydrolytic enzyme activities. Compared with untreated whole-bean tempeh, ultrasound-pretreated samples achieved significantly higher levels of peptides (up to 2.5-fold), while maintaining a moderate starch consumption profile distinct from the excessive degradation observed in dehulled tempeh. In terms of nutritional quality, ultrasound-treated samples reached approximately 55% protein bioaccessibility, bridging about 37% of the digestibility gap between whole-bean and dehulled tempeh, while preserving a low estimated glycemic index (∼49.7) substantially below that of dehulled tempeh (67.2). Among the conditions tested, 20 min of ultrasound provided the best overall performance, balancing structural disruption, fermentation efficiency, and nutrient retention without the adverse effects of excessive cavitation or complete dehulling. These findings establish ultrasound as a promising non-thermal pretreatment for producing nutritionally balanced whole-bean tempeh, offering a pathway for the high-value utilization of whole pulses in fermented functional foods.

## CRediT authorship contribution statement

**Yaohua Qin:** Writing – original draft, Investigation, Formal analysis, Data curation. **Xinyu Qiang:** Investigation. **Kun Wang:** Validation. **Jie He:** Resources. **Haijun Wang:** Methodology. **Wei Li:** Supervision. **Xin Rui:** Writing – review & editing, Supervision, Resources, Funding acquisition, Conceptualization.

## Funding

This work was co-financed by the 10.13039/501100012226Fundamental Research Funds for the Central Universities (KYQYCX2026003) and 10.13039/501100001809National Natural Science Foundation of China (32372440). The authors would also like to acknowledge a Project Funded by the 10.13039/501100012246Priority Academic Program Development of Jiangsu Higher Education Institutions (PAPD).

## Declaration of competing interest

The authors declare that they have no known competing financial interests or personal relationships that could have appeared to influence the work reported in this paper.
